# Dynamic Formation and Coupled Evolution of Oxygen-Deficient Conditions at the Return-Air Corner in a Shallow-Buried Coal Seam Working Face

**DOI:** 10.1021/acsomega.6c05393

**Published:** 2026-08-21

**Authors:** Dan Zhao, Yiming Sun, Xing Cao, Zhonghui Tian

**Affiliations:** † College of Safety Science and Engineering, 669702 Liaoning Technical University, Huludao 125000, Liaoning, China; ‡ Key Laboratory of Mine Thermodynamic Disasters and Control, Ministry of Education, Liaoning Technical University, Huludao, Liaoning Province 125000, China

## Abstract

To elucidate the dynamic formation and evolution of oxygen-deficient conditions at the return-air corner during the advancement of the No. 45207 working face in the Sandaogou Coal Mine, programmed temperature-rise experiments, field tube-bundle monitoring, and three-dimensional numerical simulations were conducted. The programmed temperature-rise experiments characterized O_2_ consumption and CO generation during the low-temperature oxidation of residual coal and provided the experimental basis for defining the species source terms in the numerical model. To overcome the limitation of conventional static models in representing the continuous evolution of the working-face position and goaf geometry, a dynamic goaf model was developed in Fluent using the dynamic-mesh layering method. The model continuously updated the working-face position and goaf extent during advancement and was compared with a conventional static model. Field-monitoring results showed that the O_2_ concentration at the return-air corner decreased from approximately 20% to 3.2% as the working face advanced, with pronounced oxygen-deficient conditions developing at an advancing distance of approximately 110 m. The numerical results further indicated that the oxygen-deficient zone migrated from the deep goaf toward the return-air side and expanded rapidly when the advancing distance reached approximately 110 m. Meanwhile, the region with O_2_ concentrations of 5%–15% gradually migrated toward the working face, accompanied by CO transport and accumulation along the return-air side. Comparison with field-monitoring data showed that the dynamic-model results agreed more closely with the measured O_2_ and CO concentrations along the return-air side than those of the static model, reducing the corresponding mean absolute errors by 45.94% and 52.16%, respectively. These findings demonstrate that the dynamic model can more effectively represent the evolution of goaf geometry, leakage airflow, and gas-transport conditions induced by working-face advancement, thereby providing a basis for identifying oxygen-deficient zones in the goaf and controlling abnormal CO accumulation at the return-air corner.

## Graphical abstract

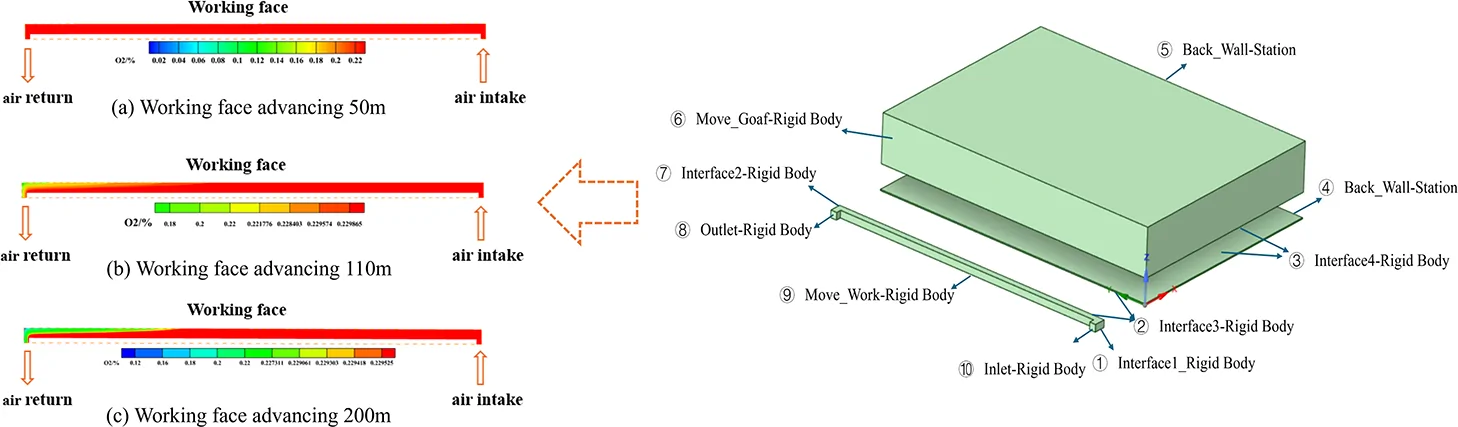

## 1 Introduction

According to the Coal Mine Safety Regulations, the oxygen concentration in the intake airflow of mining faces should not be less than 20%. A decrease in oxygen concentration may cause physiological discomfort and even hypoxic hazards to underground workers.^1,2^ The development of oxygen-deficient conditions at the return-air corner is affected by multiple factors, including residual coal oxidation, the release of oxygen-deficient gases from broken coal, atmospheric pressure fluctuations, and the evolution of overburden fractures.^3^

Previous studies have investigated the formation mechanisms of oxygen-deficient conditions at longwall working faces. Zhang^4^ reported that negative-pressure ventilation, large-scale goaf development, and atmospheric pressure fluctuations are important influencing factors. Cheng^5^ analyzed the migration and accumulation behavior of oxygen-deficient gases in the goaf and working face based on field monitoring data. Zhao^6^ demonstrated that atmospheric pressure changes can facilitate the emission of low-oxygen gases into the working face. Jin et al.^7^ investigated the mechanism and control measures of low-oxygen conditions at the return-air corner under U-type ventilation and clarified their sources and distribution characteristics. Zhang and Yang^8^ suggested that the goaf expansion scale and its “breathing effect” jointly contribute to the development of these conditions.

Gulawani et al.^9^ found that air leakage from the goaf is a major cause of oxygen-deficient gas accumulation at the return-air corner. Wasilewski^10^ reported that mine pressure varies synchronously with atmospheric pressure and that atmospheric pressure fluctuations are an important driving force for gas emission. Wang et al.^11^ indicated that oxygen consumption caused by spontaneous oxidation of residual coal plays a dominant role in oxygen depletion. Li^12^ suggested that the occurrence of low-oxygen conditions is mainly related to the location of coal seams within the N_2_–CO_2_ zone rather than to residual coal oxidation in the goaf. Guo^13^ reported that the oxidation of residual coal in adjacent and overlying goafs contributes to oxygen depletion during close-distance coal seam mining. Wang^14^ indicated that low-oxygen gas emission from adjacent goafs is another important source of oxygen deficiency.

Wang et al.^15^ investigated the distribution characteristics of oxygen concentration in the goaf using Fluent numerical simulation. Wang^16^ demonstrated that equalizing-pressure ventilation can effectively control excessive accumulation of oxygen-deficient gases at the return-air corner. Lolon et al.^17,18^ reported that gas emission intensity is closely related to decreases in atmospheric pressure. Wu^19^ established prediction models for hazardous gas concentrations and spontaneous combustion warning indices at the return-air corner.^20,21^ Qin Qinghe et al. proposed a combined equalizing-pressure ventilation technique using fans, air doors, and regulating air curtains to reduce the pressure differences at both ends of the working face and suppress harmful gas emission.

However, most existing numerical studies of low-oxygen environments in goafs are based on static geometric models and do not fully consider the dynamic structural evolution during face advancement. With continuous mining and progressive compaction of overlying strata, both the seepage field and the gas concentration field in the goaf change significantly. Therefore, a moving-boundary dynamic evolution model of the goaf was established in this study. Taking the No. 45207 working face of the Sandaogou Coal Mine as the engineering background, the spatial evolution characteristics of oxygen and CO concentration during face advancement were investigated, and the migration mechanism of oxygen-deficient conditions toward the return-air corner under the coupled effects of reduced oxygen supply and residual coal oxidation was revealed.

## 2 Mathematical Model of the Air Leakage–Oxygen-Coupling Mechanism for the Formation of Oxygen-Deficient Conditions in the Goaf

### 2.1 Basic Model Assumptions

To investigate the formation mechanism of oxygen-deficient conditions in the return corner during the advancement of the working face, and to highlight the controlling role of goaf air leakage flow and oxygen transport in the spatial distribution of oxygen concentration, the gas migration process in the goaf was reasonably simplified based on the following assumptions:

(1) The goaf was represented as a spatially heterogeneous equivalent porous medium, and the porous resistance was assumed to be locally isotropic.
(2) The gas flow satisfied the continuum assumption.
(3) The residual coal oxidation was treated using an isothermal approximation under field-observed low-temperature and slow-oxidation conditions, and its oxygen-consuming effect was represented by an O_2_ consumption source term.
(4) The gas-mixture density was evaluated using the incompressible ideal-gas model, and density variations caused by local gauge-pressure changes were neglected.

### 2.2 Continuity Equation

The mass conservation equation governing gas migration in the goaf is given as

1 $$\frac{\partial \rho }{\partial t}+\frac{\partial \left(\rho u\right)}{\partial x}+\frac{\partial \left(\rho v\right)}{\partial y}+\frac{\partial \left(\rho w\right)}{\partial z}=S_{m}$$

where ρ is the density (kg/m^3^), *t* denotes time (s), *u*, *v*, and *w* are the average gas velocities spatially per unit time (m/s), and *S*_*m*_ is the mass source term representing gas emission from the goaf (kg/(m^3^·s)).

### 2.3 Momentum Equation

The gas flow field in the goaf was further described by the momentum conservation equation

2 $$\frac{\partial \left(\rho \mathrm{v⃗}\right)}{\partial t}+\nabla \cdot \left(\rho \mathrm{v⃗}\mathrm{v⃗}\right)=-\nabla p+\nabla \cdot \left(\mu \nabla \mathrm{v⃗}\right)+{\mathrm{S⃗}}_{v}$$

where *p* denotes the pressure (Pa), μ is the dynamic viscosity (Pa·s), and ${\mathrm{S⃗}}_{v}$ represents the momentum sink term induced by the porous medium.

To represent the resistance effect of the collapsed rock mass, the porous-medium source term was defined using the Darcy–Forchheimer relation

3 $$S_{i}=-\frac{\mu }{\alpha }V_{i}-\frac{1}{2}C_{2}\rho \left|V_{j}\right|V_{j}$$

where μ is the dynamic viscosity coefficient (Pa·s), *k* is the permeability of the goaf (m^2^), and *C*_2_ is the inertial resistance coefficient (1/*m*). According to the Blake–Kozeny equation, the empirical formula for non-Darcy flow is defined as^22^

4 $$k=\frac{D_{m}^{2}n^{3}}{150{\left(1-n\right)}^{2}}$$

5 $$C_{2}=\frac{3.5\left(1-n\right)}{D_{m}n^{3}}$$

where *D*_m_ is the average particle size of the falling rock in the goaf and *n* is the porosity.

### 2.4 Mass Equation for Gas Composition

The evolution of gas composition in the goaf was described by the species transport equation

6 $$\frac{\partial \left(\rho Y_{i}\right)}{\partial t}+\nabla \cdot \left(\rho \mathrm{V⃗}Y_{i}\right)=-\nabla \cdot {\mathrm{J⃗}}_{i}+F_{i}+Q_{i}$$

where *Y*_*i*_ is the mass fraction of gas component *i*(−), ${\mathrm{J⃗}}_{i}$ is the diffusion flux of species *i* (kg/(m^2^·s)), *F*_*i*_ is the net production rate of the redox reaction (kg/(m^3^·s)), and *Q*_*i*_ is the rate of oxygen consumption (mol/(m^3^·s)).

According to Fick’s law, the diffusion flux can be expressed as

7 $$J_{i}=-\rho D_{i,eff}\nabla Y_{i}$$

where *D*_*i*,eff_ denotes the effective diffusion coefficient.

### 2.5 Oxygen Consumption Source Term Model

The influence of residual coal oxidation on oxygen distribution in the goaf was considered by introducing an oxygen sink term into the species transport equation. In this way, oxygen depletion induced by low-temperature coal oxidation could be incorporated into CFD calculations. According to the programmed temperature-rise experiments, the oxygen consumption rate was obtained as

8 $$V_{O_{2}}^{0}\left(T\right)=\frac{QC_{1}}{V_{m}}\ln\frac{C_{1}}{C_{2}}$$

Meanwhile, the associated CO generation rate was calculated by

9 $$V_{CO}^{0}\left(T\right)=\frac{V_{O_{2}}^{0}\left(T\right)C_{CO}}{C_{O_{2}}\left[1-e^{-V_{O_{2}}^{0}\left(T\right)V_{m}/\left(QC_{O_{2}}\right)}\right]}$$

where $V_{O_{2}}^{0}\left(T\right)$ and *V*_CO_^0^(*T*) denote the oxygen consumption rate and CO generation rate at temperature *T*, respectively, *Q* is the inlet airflow rate (m^3^/s), *V*_m_ is the volume of the coal sample (m^3^), *C*_1_ is the oxygen molar concentration at the inlet (mol/m^3^), *C*_2_ is the oxygen molar concentration at the outlet (mol/m^3^), *C*_CO_ is the CO molar concentration at the outlet (mol/m^3^), and *C*_O_2__ is the oxygen molar concentration in the gas mixture (mol/m^3^).

### 2.6 Permeability Evolution of the Goaf

According to the O-ring effect, the spatial distribution of the bulking factor in the goaf can be expressed as follows^23^

10 $$K_{p}\left(x,y\right)=K_{p,\min}+\left(K_{p,\max}-K_{p,\min}\right)\exp\left\{-\alpha_{x}d_{x}\left[1-\exp\left(-\xi \alpha_{y}d_{y}\right)\right]\right\}$$

where *K*_*p*_(*x*,*y*) is the bulking factor distribution function in the goaf, *K*_*p*,max_ and *K*_*p*,min_ represent the maximum bulking factor at the initial caving stage and the minimum bulking factor after compaction, respectively, α_*x*_ and α_*y*_ are attenuation coefficients along the strike and dip directions, *d*_*x*_ and *d*_*y*_ denote the distances from any point (*x*, *y*) to the surrounding solid boundary and working face boundary, and ξ is the shape-control coefficient of the O-ring distribution model.

In this study, the structural parameters of the goaf were linked to the bulking behavior of the broken rock mass. Accordingly, the height of the caving zone and the porosity distribution were determined by the following expressions^24,25^

11 $$H=\frac{MK_{p}\left(x,y\right)}{K_{p,\max}-1}$$

12 $$\varepsilon =1-\frac{1}{K_{p}\left(x,y\right)}$$

where *H* is the height of the caving zone (m) and *M* is the mining height of the working face (m).

## 3 Numerical Model of the Dynamic Evolution of Oxygen-Deficient Conditions in the Goaf

### 3.1 Overview of the 45207 Working Face

Figure 1 presents a schematic diagram of the working face. The 45207 working face is located in the No. 5–2 coal seam. Topographically, the mining area shows clear spatial variability in surface elevation, ranging from about 1008.5 to 1397.0 m above sea level. The terrain is relatively higher in the central-western sector and gradually decreases toward the surrounding areas, particularly the southeastern part. Across most of the study area, surface elevations mainly fall within 1200–1250 m, and the local relief is generally 100–150 m. The overall maximum elevation difference reaches approximately 388.5 m, with the highest point located in the central zone and the lowest point near the southeastern confluence area. The 45207 working face adopts a U-type ventilation system.

**Figure 1:**
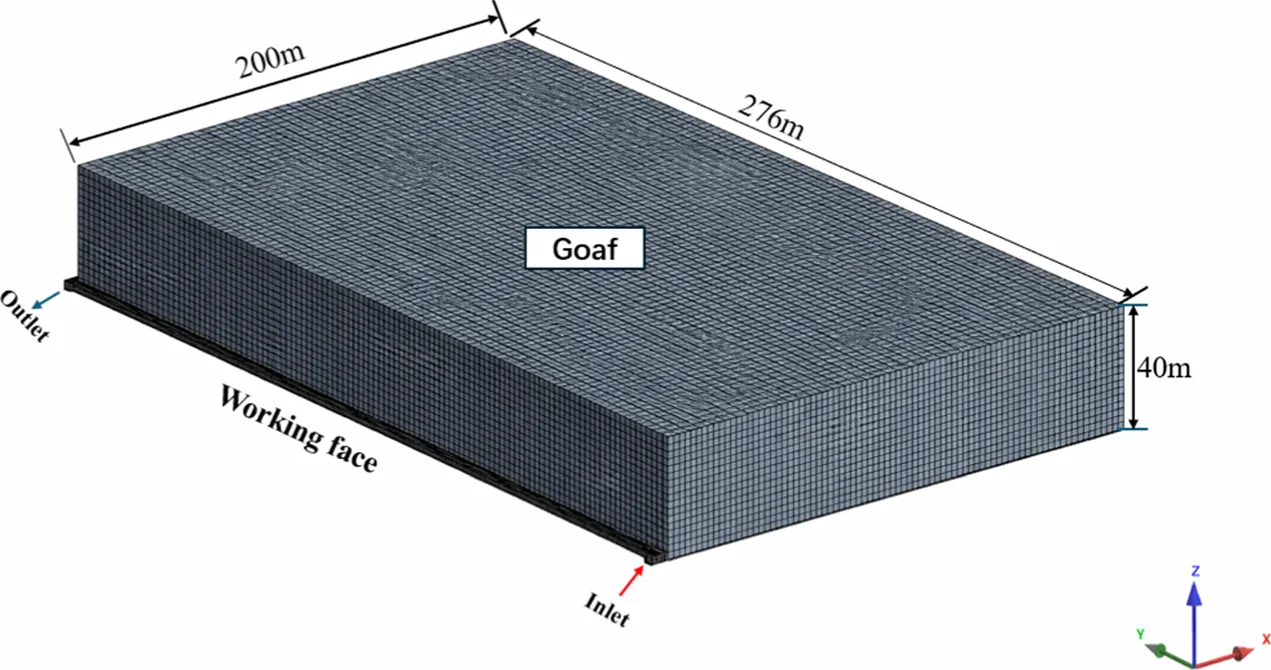
Schematic diagram of the geometric model.

### 3.2 Dynamic Evolution of Low-Oxygen Conditions in the Goaf

In Figure 2, as the working face continuously advances, the extent of the goaf and the positions of its boundaries change accordingly, resulting in corresponding variations in the air-leakage-induced oxygen supply and gas-species transport within the goaf. To reproduce the continuous evolution of the computational domain and gas concentration fields during working face advancement, the dynamic mesh technique available in Fluent was employed to simulate the movement of the working face. Considering the relatively regular geometry of the computational domain and the use of hexahedral cells, the layering method was selected for dynamic mesh updating. The collapse factor and split factor were set to 0.4 and 0.2, respectively, while the initial mesh layer height was set to 1 m during mesh generation. When the mesh layers adjacent to the moving boundary satisfied the prescribed splitting or collapsing criteria, mesh layers were automatically added or removed by Fluent. In this manner, the movement of the working face and the dynamic expansion of the goaf computational domain were reproduced.

**Figure 2:**
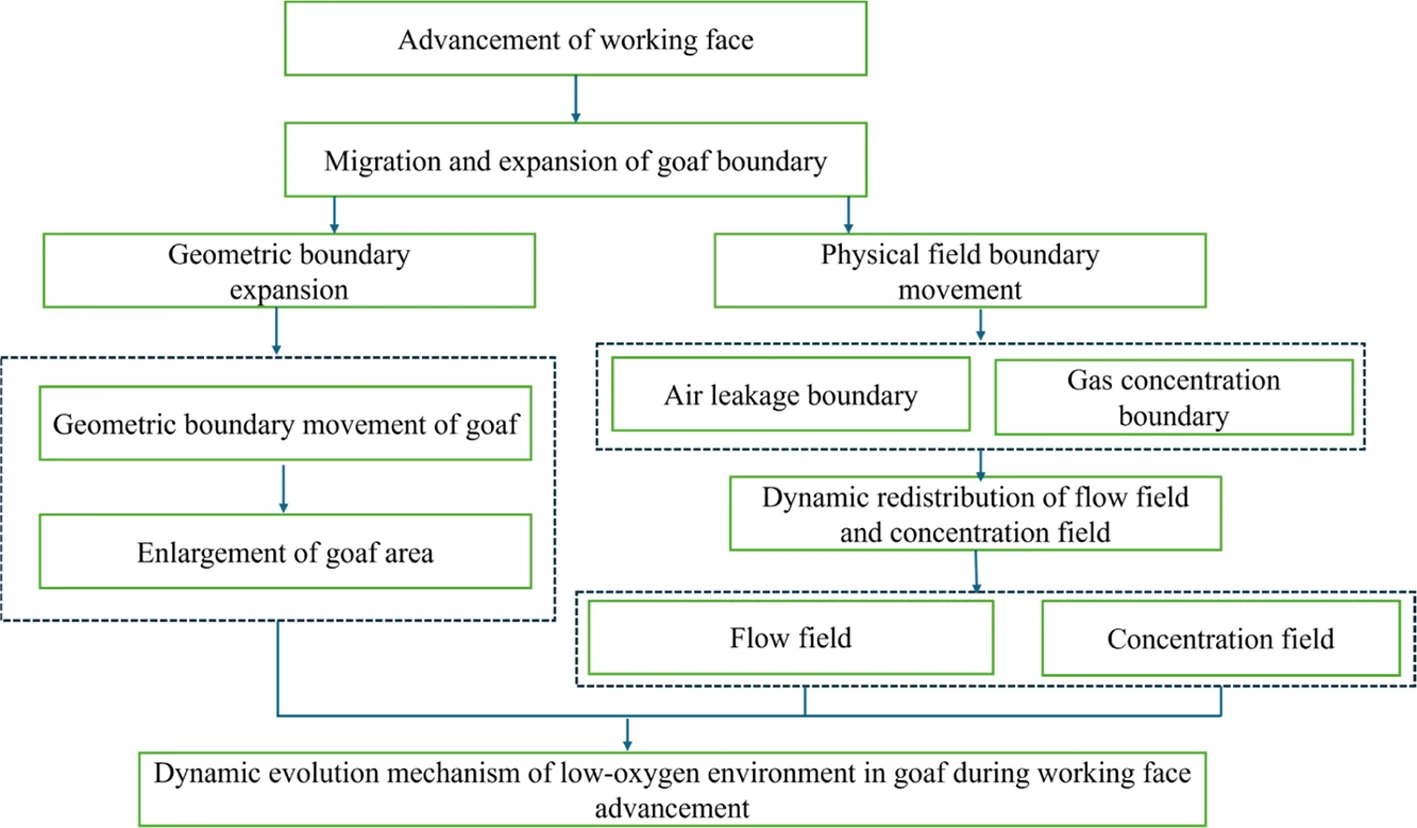
Dynamic evolution of the low-oxygen region in the goaf.

The motion of the moving boundary was prescribed using a profile file, in which the boundary was translated at a constant velocity of (5.79 × 10^–5^) along the negative (*X*)-direction. In addition, user-defined functions were employed to dynamically control the key goaf parameters, including porosity, viscous resistance, inertial resistance, and oxygen-consumption rate.

Meanwhile, continuous face advancement causes dynamic changes in both the geometric scale of the goaf and the internal physical-field distribution. The interaction between newly formed and previously compacted regions results in a coupled evolution of the internal flow structure and the oxygen concentration field. In addition, as the goaf area expands with face advancement, the size of the computational domain also changes accordingly (Figure 3). Therefore, a dynamic mesh adjustment strategy was adopted to simulate the progressive growth of the goaf.

**Figure 3:**
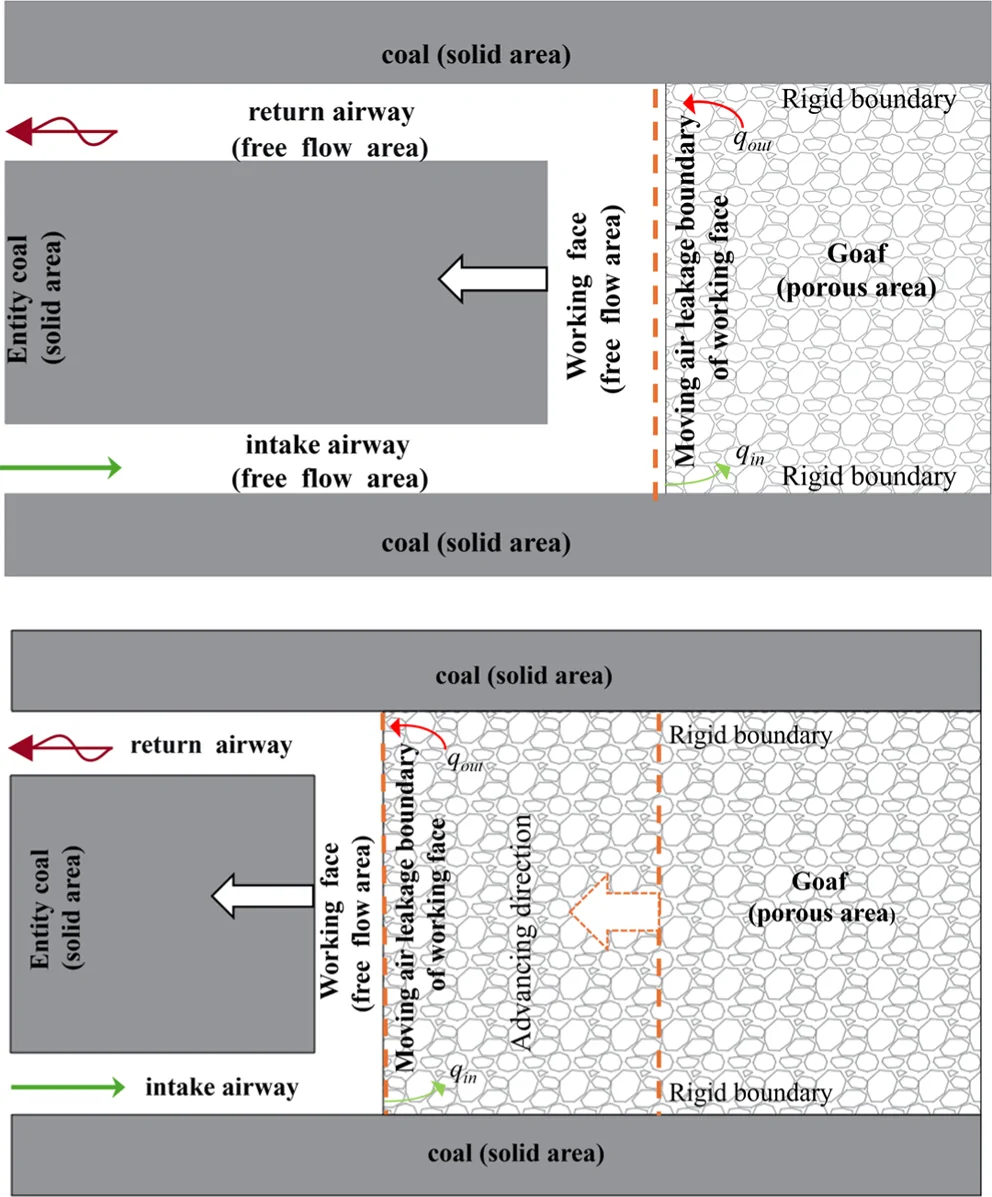
Change of computing domain during the dynamic advancement of goaf.

### 3.3 Boundary Conditions and Model Parameters

As mining proceeds, the near-face leakage boundary shifts continuously toward the advancing direction, accompanied by the gradual enlargement of the goaf. To account for this evolving boundary condition, ANSYS Fluent was used with a staged advancing approach, whereby the computational region was reconstructed stepwise as the face moved forward.

Specifically, geometric models corresponding to different advancing distances were established to achieve a progressive expansion of the computational domain during face advancement. This approach enables the dynamic evolution of low-oxygen conditions to be described under the coupled effects of goaf structural changes and air leakage migration.

The corresponding calculation parameters and boundary conditions are given in Tables 1 and 2.

**Table 1:** Main Parameters of the Calculation Model

model part	geometric dimensions
ventilation patterns	U type
air intake roadway	length 3 m × width 3 m × height 3 m
air return roadway	length 3 m × width 3 m × height 3 m
working face	length 276 m × width 6 m × height 3 m
goaf	length 200 m × width 276 m × height 40 m
float coal	length 200 m × width 276 m × height 0.5 m

**Table 2:** Numerical Simulation Parameters

boundary condition	parameters
inlet boundary condition	(velocity-inlet) 1.2 m/s
outlet boundary condition	outflow
viscous and inertial resistance	use PROFILE macro definition
air mining area	porous medium
oxygen consumption rate/(kg/(m^3^·s))	according to experimental and actual conditions
working face	fluids

## 4 Oxygen Consumption and Gas Generation during the Low-Temperature Oxidation of Residual Coal

### 4.1 Programmed Temperature Rise Experimental System

Temperature-programmed oxidation experiments were conducted on coal samples collected at the 45207 working face of the Sandaogou Coal Mine using a programmed temperature rise experimental system, and the generated gas components were analyzed by gas chromatography (Figure 4).

**Figure 4:**
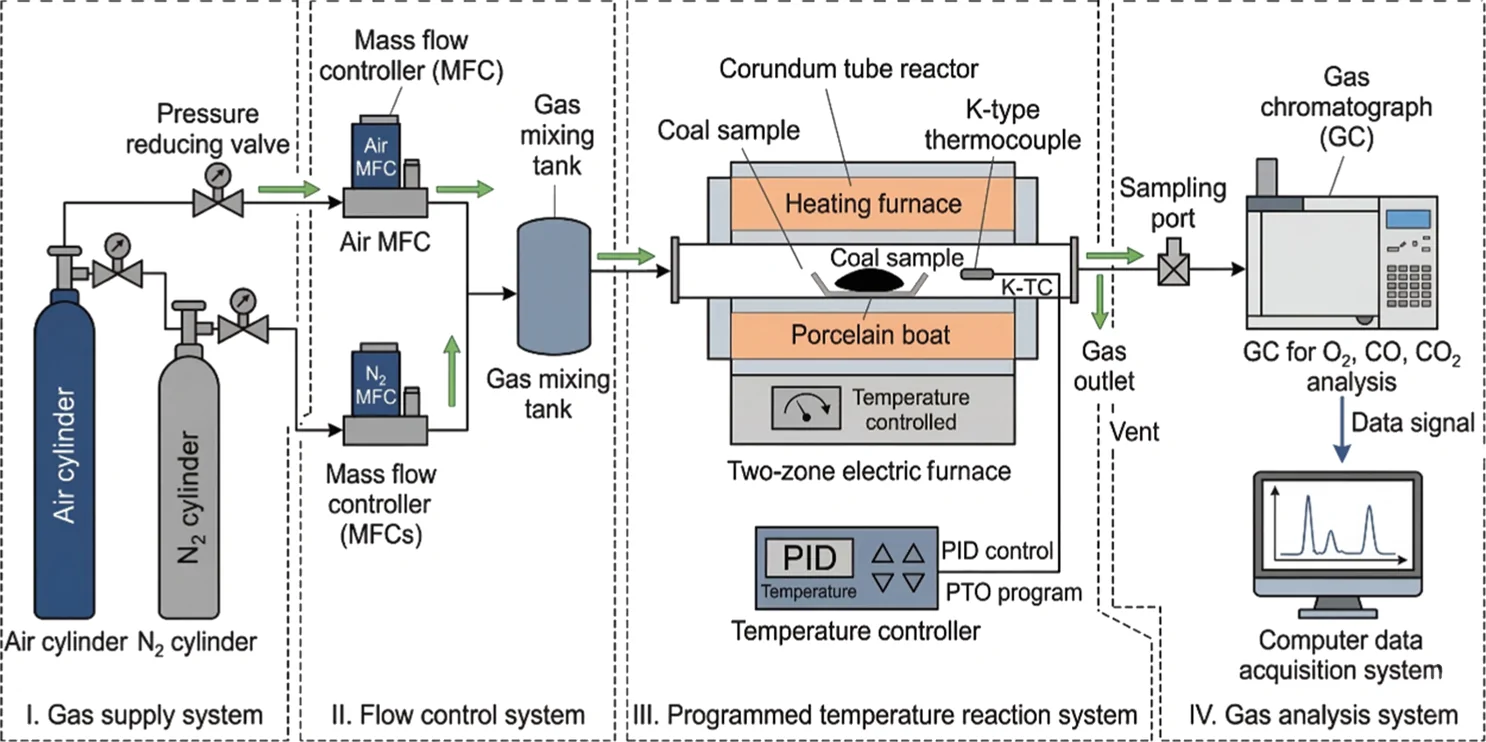
Programmed temperature-raising box and gas chromatography coupling equipment.

The detailed experimental procedure was as follows: (1) fresh coal samples were sieved to obtain particle sizes ranging from 8 to 24 mm. Across all experimental runs, a cumulative total of approximately 800 g of coal sample was used. (2) Before each run, the prepared coal sample was loaded into the sample tank, and the airtightness of the experimental system was checked. Dry air was then introduced for 5 min to purge the residual gases. (3) The gas chromatograph was calibrated prior to gas analysis. (4) The inlet O_2_ concentrations were set to 21%, 18%, 16%, 12%, 9%, and 6% by mixing O_2_ and N_2_ in controlled proportions. (5) The programmed temperature-rise experiment was initiated at 30 °C. The heating rate was set to 1 °C/min from 30 to 100 °C, with gas sampling at intervals of 10 °C, and to 2 °C/min from 100 to 300 °C, with gas sampling at intervals of 20 °C. At each sampling temperature, the system was maintained at a constant temperature for 25 min before the gas composition was analyzed by gas chromatography. (6) The inlet O_2_ concentration was monitored during each experimental run, which was considered valid when the concentration variation remained below 0.1%. The procedure was repeated at each specified inlet O_2_ concentration.

### 4.2 Oxygen Consumption and Gas Generation Characteristics of Residual Coal

Figure 5 presents the variation of outlet oxygen concentration with coal temperature under different oxygen concentration conditions. As the temperature increases, the oxygen volume fraction decreases gradually in all cases, with a pronounced accelerated oxygen consumption stage observed within the temperature range of 120–180 °C. Under higher oxygen concentration conditions (21% and 18%), oxygen consumption remains relatively slow at low temperatures (<120 °C) but decreases rapidly after entering the intermediate-temperature stage.

**Figure 5:**
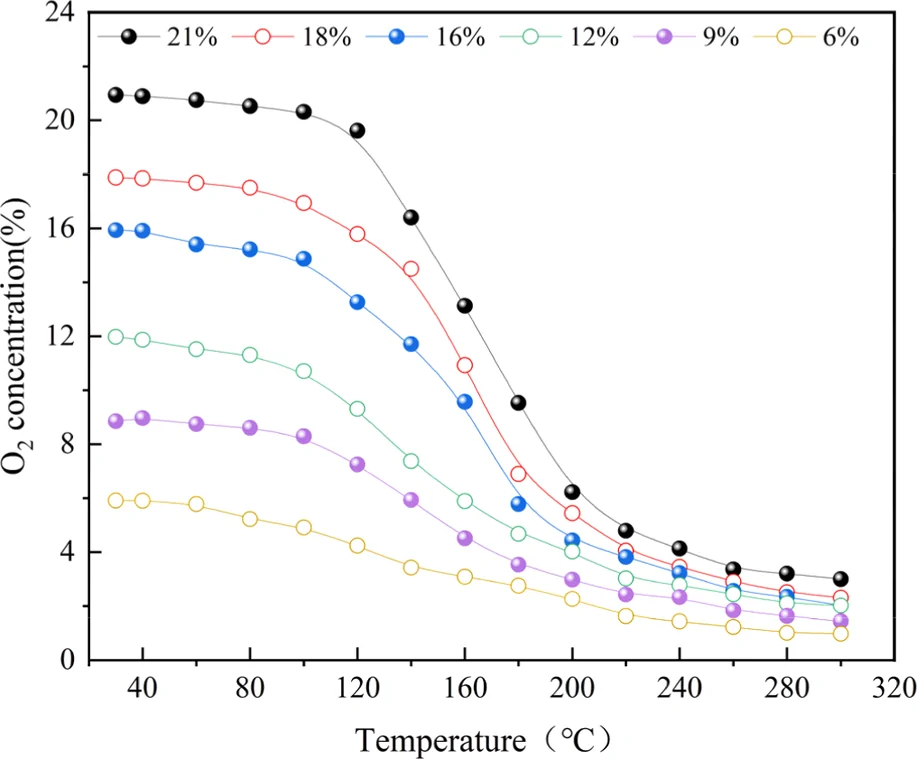
Variation law of the O_2_ concentration.

These results indicate that residual coal oxidation can continuously consume oxygen under sustained oxygen supply conditions, which plays an important role in the formation of low-oxygen environments in the goaf. Meanwhile, CO is generated simultaneously with oxygen consumption and can serve as an indicator gas for evaluating the oxidation state of the residual coal.

Figure 6 illustrates the variation of CO concentration with temperature, showing a typical staged growth pattern during residual coal oxidation. At the low-temperature stage (<80 °C), the CO concentration remains very low, indicating weak oxidation activity. Within the temperature range of 80–150 °C, the CO concentration increases markedly, suggesting the transition to an accelerated oxidation stage. When the temperature exceeds 150 °C, the CO concentration rises rapidly and exhibits a near-exponential growth trend.

**Figure 6:**
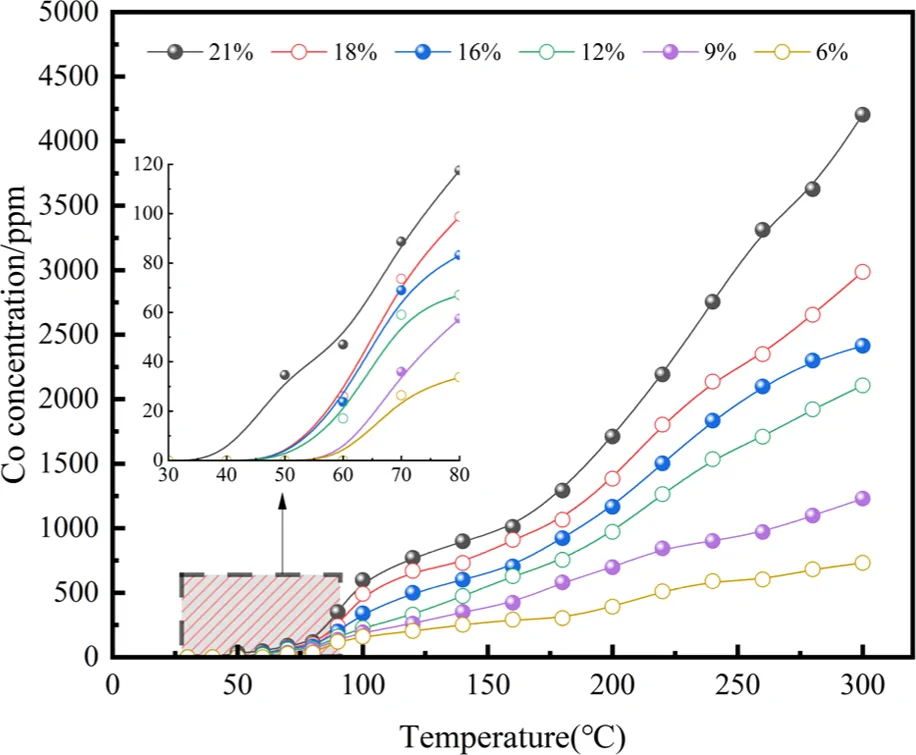
Variation law of the CO concentration.

This increase becomes more pronounced under higher oxygen concentration conditions. At 300 °C, the CO concentration exceeds 4000 ppm under 21% oxygen concentration but is only about 700 ppm under 6% oxygen concentration, indicating the strong dependence of CO generation on oxygen supply. These results demonstrate that CO concentration can serve as an effective indicator of residual coal oxidation and further confirm that residual coal oxidation contributes to oxygen consumption and promotes the development of low-oxygen environments in the goaf.

The oxygen consumption rate calculated from eqs 8 and 9 is shown in Figure 7.

**Figure 7:**
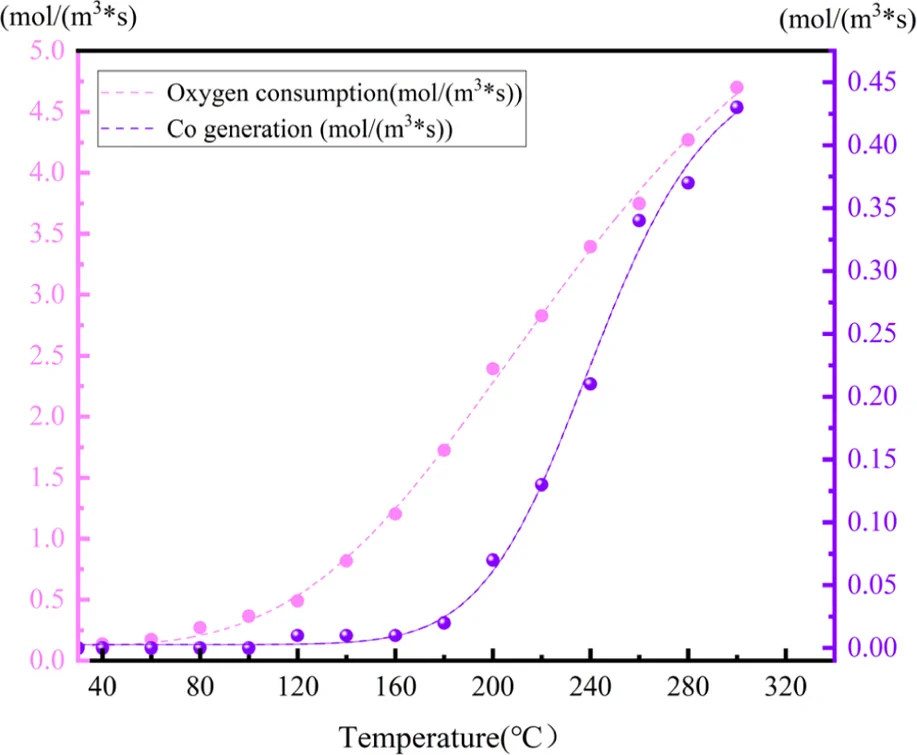
CO generation rate and oxygen consumption rate as functions of temperature.

During the programmed temperature increase process, residual coal oxidation exhibits typical staged characteristics of oxygen consumption and CO generation. The oxygen consumption rate increases continuously with temperature, whereas the CO generation rate increases slowly at low temperatures but rises markedly at higher temperatures, showing typical lagging formation behavior.

These results indicate that oxygen is preferentially consumed during the early oxidation stage while CO is mainly generated during the accelerated oxidation stage. Under continuous oxygen supply conditions, such as air leakage in the goaf, this process promotes oxygen depletion and CO accumulation, providing experimental support for the formation mechanism of low-oxygen environments in the goaf.

## 5 Results and Discussion

### 5.1 Spatial Evolution of Oxygen Concentration in the Goaf during Face Advancement

Figure 8 shows the distribution of oxygen concentration in the goaf under different advancing distances. As the working face advances, the goaf continuously expands in both the spatial extent and depth. Meanwhile, the compaction degree in the middle and deep regions gradually intensifies, and fracture channels progressively shrink, resulting in shortened air leakage pathways and a reduced oxygen supply capacity within the goaf.

**Figure 8:**
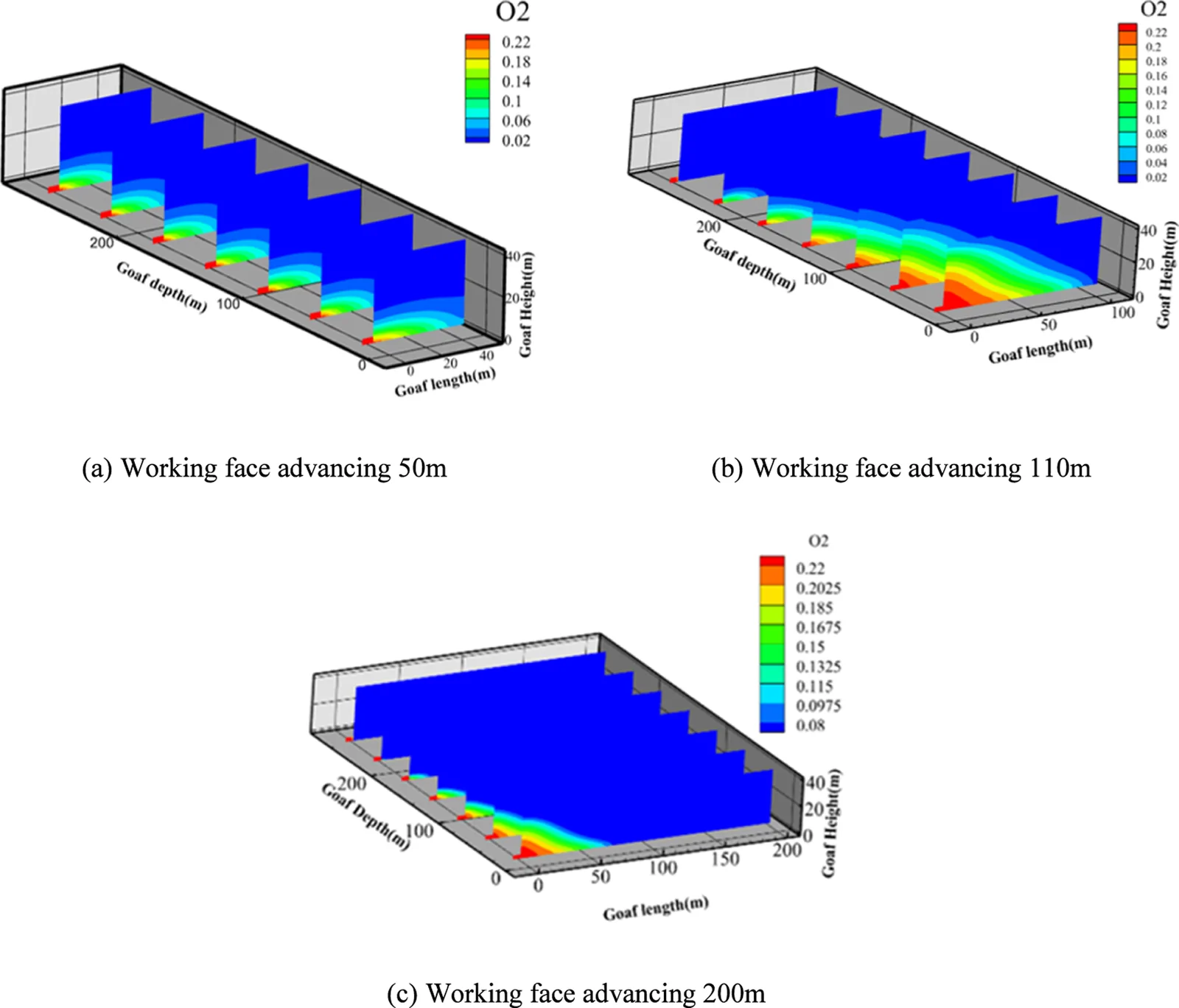
3D oxygen distribution in goaf along the work surface direction.

### 5.2 Spatial Distribution Characteristics of Oxygen Concentration in the Return-Air Corner

Figure 9 illustrates how oxygen concentration near the return-air corner changes with increasing advancing distance. The oxygen field in the goaf is nonuniform, and the oxygen-rich area is more extensive on the intake side than on the return side. At the early advancing stage (50 m), the goaf is not yet fully developed, so oxygen replenishment from leakage airflow remains effective, and the oxygen concentration is relatively high.

**Figure 9:**
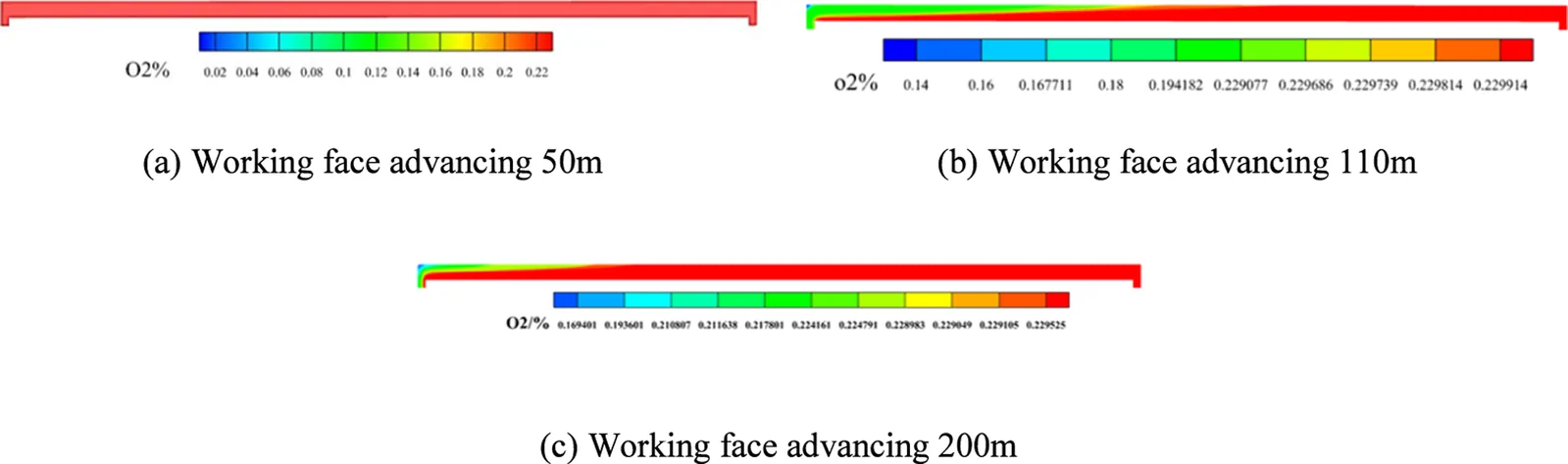
Oxygen concentration at the return-air corner of the working face.

As the advancing distance increases to approximately 110 m, the middle region of the goaf becomes gradually compacted, leading to a reduced oxygen supply capacity. Consequently, the low-oxygen zone expands and migrates toward the return-air corner. With further advancement, the oxygen concentration field gradually became stable. This evolution pattern agrees well with the programmed temperature increase experiments and confirms the reliability of the proposed dynamic goaf model.

### 5.3 Migration of Low-Oxygen Zones in the Goaf with Advancing Distance

As shown in Figure 10, at the *z* = 1.5 m section, the oxygen concentration distribution along the depth direction varies significantly under different advancing distances. The characteristic oxygen concentration boundaries (0.15 and 0.05) gradually migrate toward the working face as the advancing distance increases, indicating that the low-oxygen-source region moves forward with the structural evolution of the goaf.

**Figure 10:**
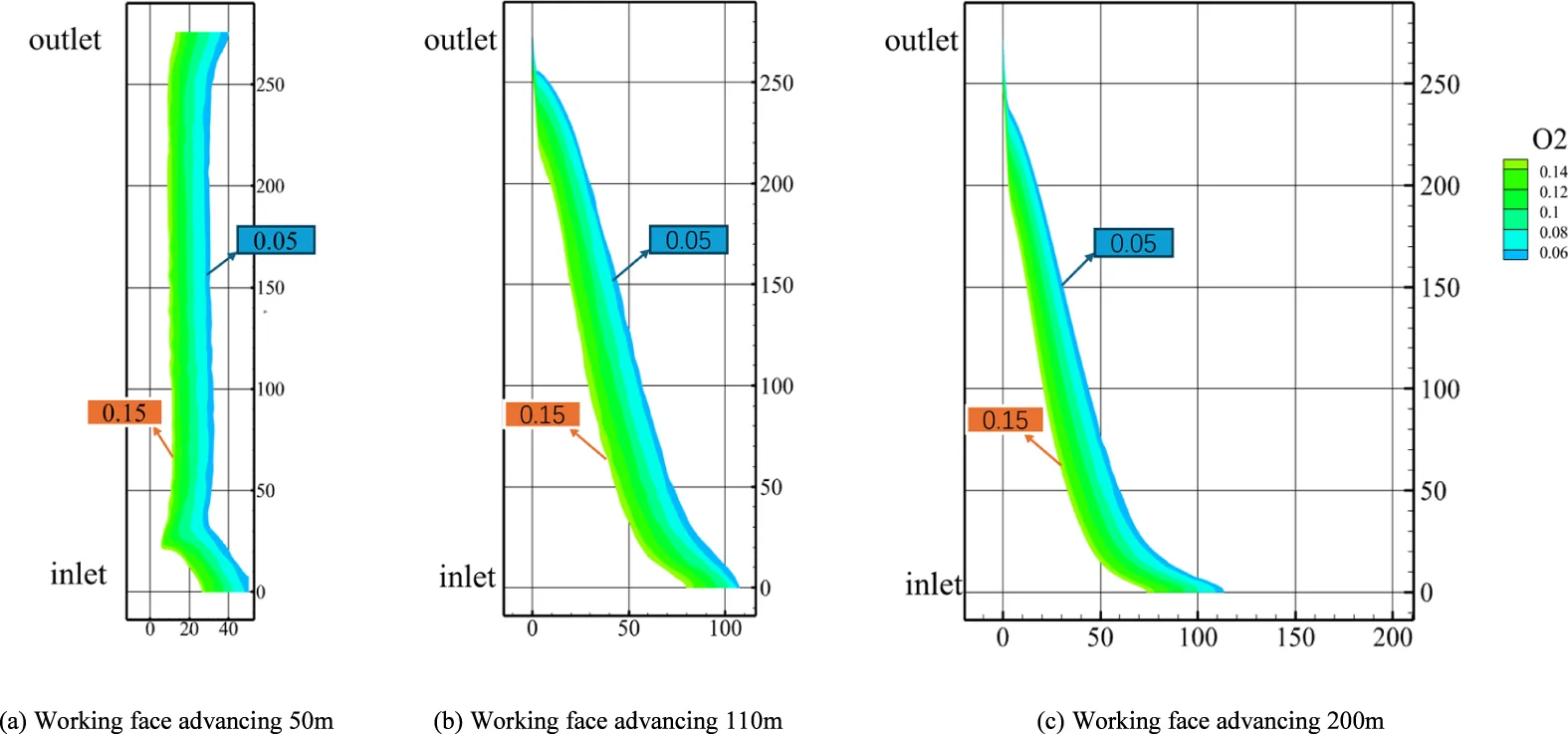
Distribution of oxygen concentration self-ignition area in goaf (*z* = 1.5 m).

With the progressive compaction of the middle and deep regions of the goaf, air leakage pathways become shortened, and oxygen supply capacity decreases. Meanwhile, continuous oxygen consumption caused by residual coal oxidation further promotes the forward migration of the low-oxygen zone, providing important conditions for the formation of low-oxygen environments at the return-air corner.

### 5.4 Spatial Distribution Characteristics of CO Concentration in the Return-Air Corner

During actual mining operations at the Sandaogou Coal Mine, CO concentration exceedances frequently occur. However, no indicator gases of coal spontaneous combustion were monitored, and no significant temperature increase was observed in the goaf, making the source of CO at the return-air corner unclear.^26–28^

As shown in Figure 11, with increasing advancing distance, the geometric boundary of the goaf expands continuously, while the internal compaction degree gradually increases, resulting in reduced oxygen supply capacity from air leakage. Meanwhile, continuous oxygen consumption caused by residual coal oxidation further promotes CO generation. Under the combined effects of these factors, the low-oxygen zone gradually migrates toward the return-air side and ultimately affects the gas distribution in the return-air corner, which is an important mechanism for the formation of the low-oxygen environment in this region.

**Figure 11:**
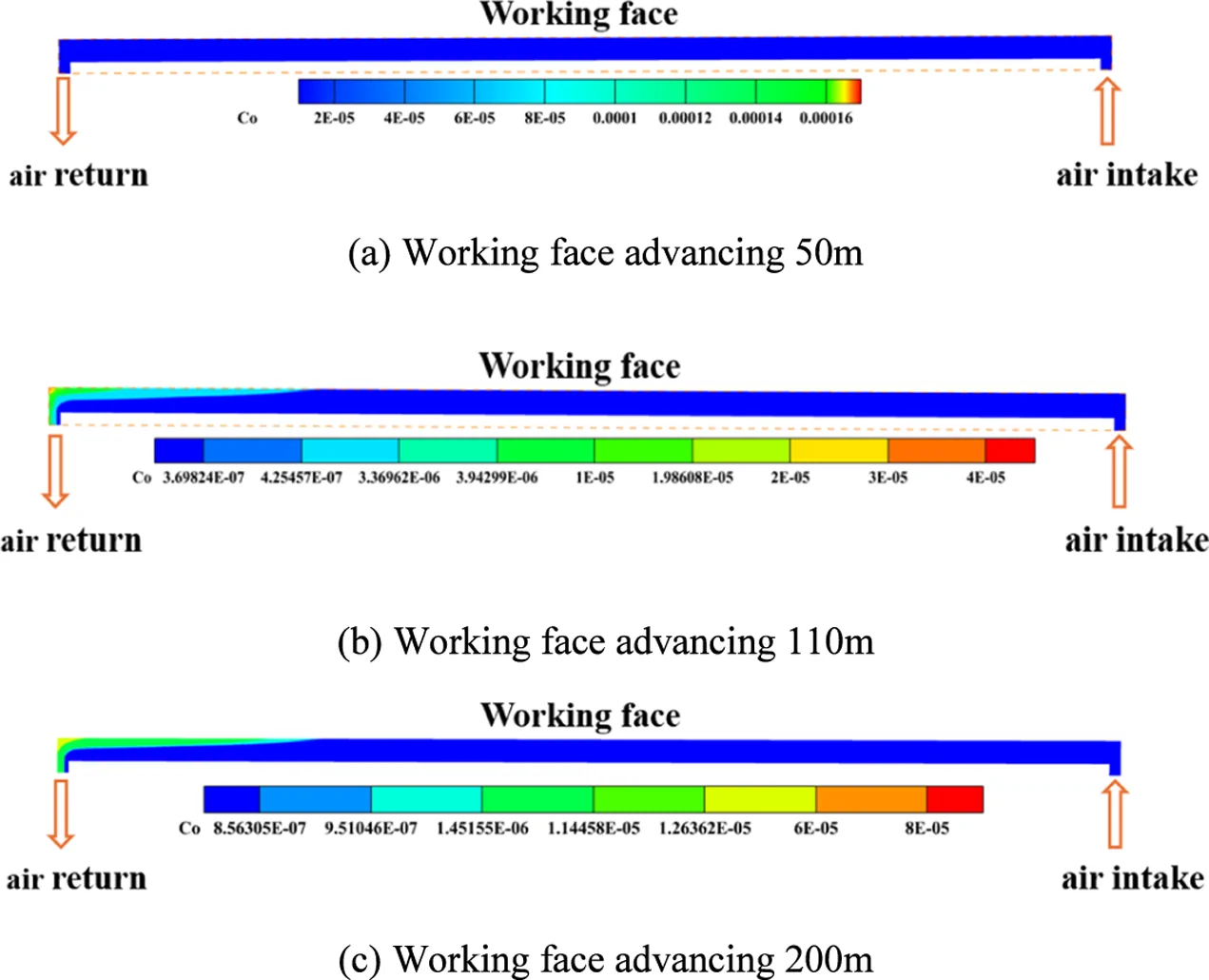
Carbon monoxide concentration field in the goaf.

### 5.5 Comparison of O_2_ Concentrations Predicted by the Dynamic-Mesh and Static Models

To facilitate a direct comparison of O_2_ concentration variations at different locations, several monitoring points near the return-air corner were selected, and their corresponding coordinates are listed in Table 3.

**Table 3:** O_2_ Concentrations at Different Monitoring Points

monitoring point	coordinates	monitoring point	coordinates
A	(2,276,0.4)	B	(4,276,0.4)
C	(6,276,0.4)	D	(2,274,0.4)
E	(4,274,0.4)	F	(6,274,0.4)
G	(2,272,0.4)	H	(4,272,0.4)
I	(6,272,0.4)	J	(2,270,0.4)
K	(4,270,0.4)	L	(6,270,0.4)

Figure 12 presents a local comparison at 12 corresponding monitoring locations near the return-air corner when the working face had advanced to 110 m. Pronounced oxygen-deficient conditions had developed near the return-air corner at this stage. The dynamic model reproduced the measured O_2_ concentrations and their spatial variation more closely, whereas the static model generally overestimated the O_2_ concentrations. This tendency is primarily associated with the inability of the static model to account for the continuous evolution of the computational domain during the working-face advancement. For this local data set, the MAE and RMSE of the dynamic model were 2.00 and 2.02 percentage points, respectively, compared with 4.53 and 4.54 percentage points for the static model. Based on the corresponding unrounded values, the dynamic model reduced the MAE and RMSE by 55.82% and 55.47%, respectively. These results demonstrate that continuously updating the working face position and goaf boundary enables the model to better capture the effects of air-leakage flow redistribution on the O_2_ supply and the development of oxygen-deficient conditions near the return-air corner.

**Figure 12:**
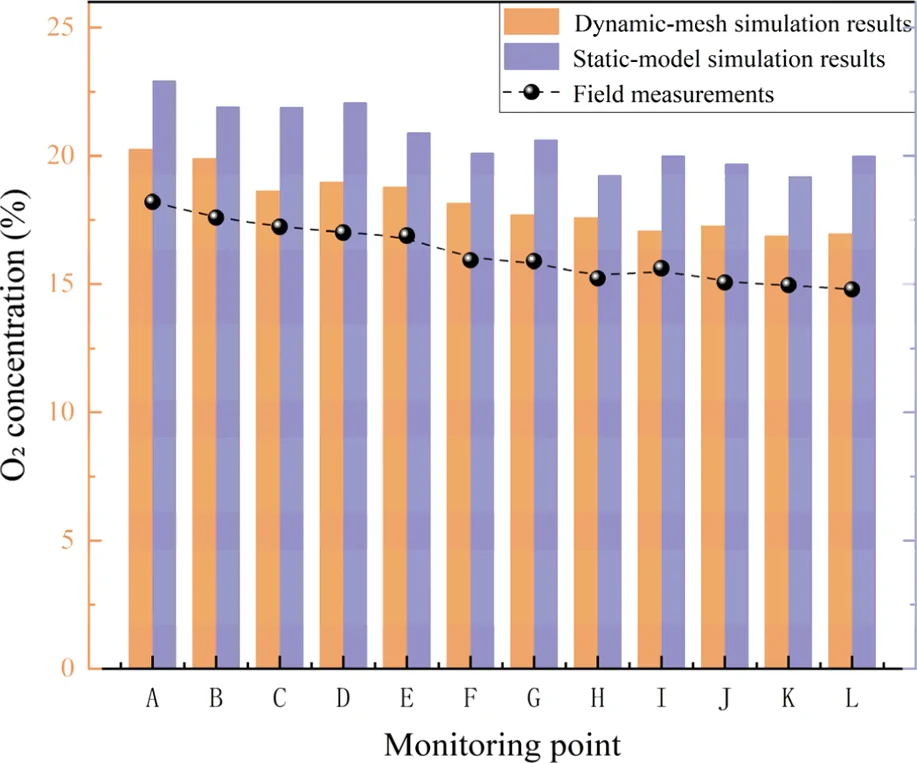
Comparison of O_2_ concentrations obtained from the dynamic-mesh model, static model, and field measurements at different monitoring points.

### 5.6 Validation with Field Monitoring Data

Field data were obtained from the tube-bundle monitoring system during the mining of the fully mechanized working face at the Sandaogou Coal Mine. The monitoring points were arranged along the return-air side of the goaf at intervals of approximately 50 m. The system automatically collected gas samples and analyzed their compositions at intervals of ∼10 min. For model validation, data were selected from periods characterized by stable monitoring system operation, continuous working face advancement, and the absence of obvious ventilation disturbances.

As shown in Figure 13, the measured O_2_ concentration decreased with increasing goaf depth, whereas the CO concentration initially increased and subsequently decreased. In contrast to the local comparison near the return-air corner presented in Figure 12, the quantitative evaluation in Figure 13 was based on all valid monitoring data collected at different goaf depths along the return-air side. At the corresponding goaf depths and stages of working face advancement, the predictions of the dynamic and static models were compared separately with the field measurements. For the O_2_ concentration, the dynamic model yielded an *R* ^2^ of 0.96 and an MAE of 1.07 percentage points, whereas the static model yielded an *R* ^2^ of 0.82 and an MAE of 1.97 percentage points. For the CO concentration, the *R* ^2^ and MAE of the dynamic model were 0.92 and 1.66 ppm, respectively, compared with 0.65 and 3.47 ppm for the static model. These results indicate that the dynamic model achieved better agreement with the field measurements for both O_2_ and CO and more accurately captured the spatial variation in gas concentrations along the return-air side during working face advancement.

**Figure 13:**
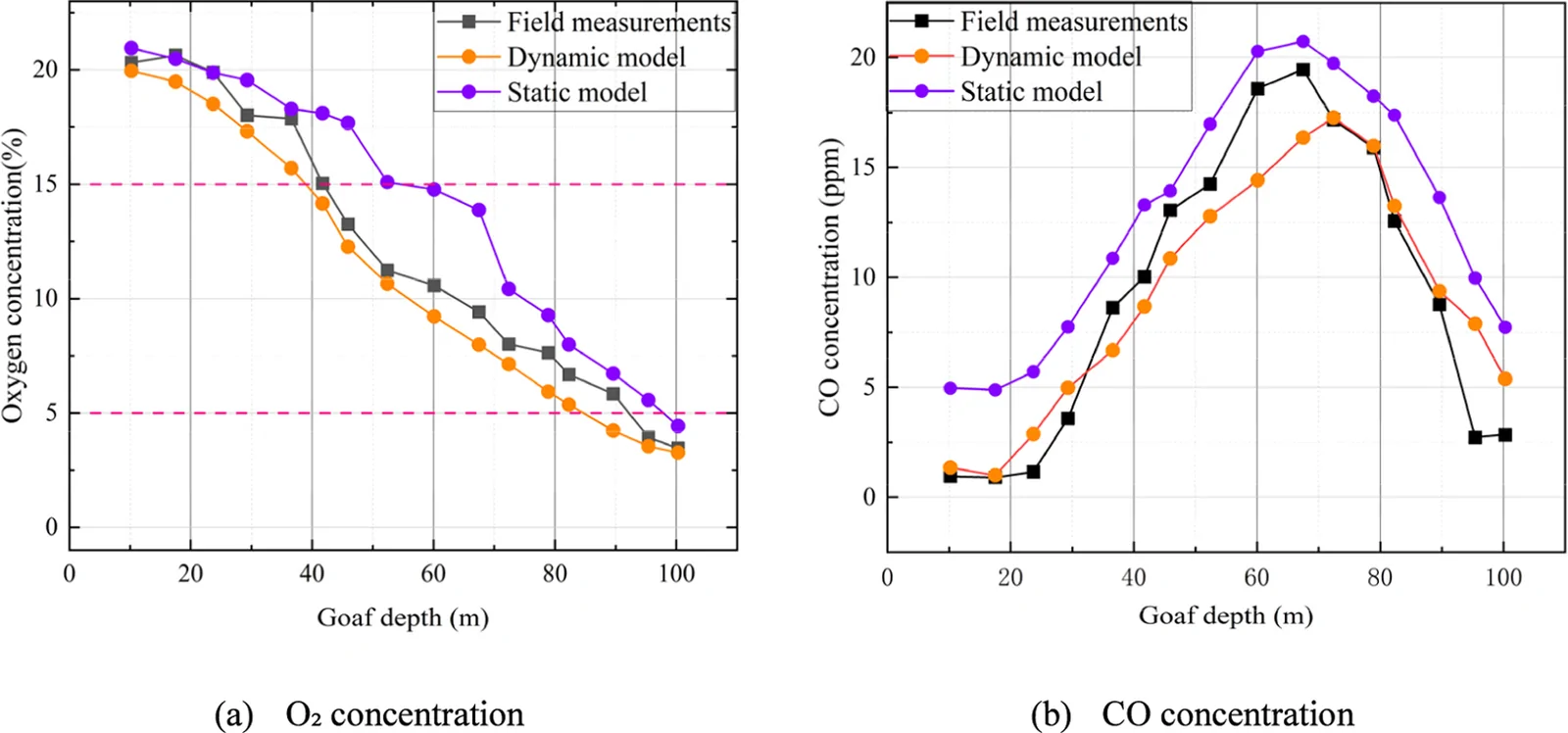
Comparison of measured and simulated O_2_ and CO concentrations along the return-air side.

## 6 Conclusion

(1) As the working face advanced, the O_2_ concentration at the return-air corner decreased from approximately 20% to 3.2%. Oxygen-deficient conditions became markedly more pronounced at an advancing distance of approximately 110 m, indicating a critical stage in the formation of these structures.
(2) The dynamic model showed that the oxygen-deficient zone migrated from the deep goaf toward the return-air side, while the region with O_2_ concentrations of 5%–15% moved toward the working face. Meanwhile, CO was transported and accumulated along the return-air side. This evolution was jointly influenced by changes in goaf structure and compaction, leakage-air O_2_ supply, and continuous O_2_ consumption by residual coal.
(3) Within the monitored range along the return-air side, the MAEs of the simulated O_2_ and CO concentrations obtained using the dynamic model were 45.94% and 52.16% lower, respectively, than those obtained using the static model. The dynamic-model results agreed more closely with the field measurements, indicating that the model better represented changes in leakage airflow and gas transport during working face advancement.
